# The effect of seeing a family physician on the level of glycosylated hemoglobin (HbA1c) in type 2 Diabetes Mellitus patients

**DOI:** 10.1186/2251-6581-12-2

**Published:** 2013-01-05

**Authors:** Ali Reza Mahdavi, Koorosh Etemad, Muhiuddin Haider, Seyed Mohammad Alavinia

**Affiliations:** 1 Ministry of Health and Education, Center for non-communicable disease control, Tehran, Iran; 2 SPH Building, University of Maryland, School of Public Health, College Park, MD, 20742, USA; 3 North Khorasan University of Medical Sciences, Zoonosis Research Center and School of Medicine, Bojnurd, Iran

**Keywords:** Type 2 diabetes, HbA1c, Family physician

## Abstract

**Background:**

Glycosylated hemoglobin (HbA1c) in diabetic patients reflects the average blood glucose level, and will not be affected by variability in blood glucose in short time. Regular care of patients by medical staff could effectively control glycemic situation. The aim of this study was to assess the effect of medical care by general physicians on glycemic control by measuring of HbA1c.

**Methods:**

In order to assess the effectiveness of National program for diabetes control and prevention in Iran, we compare HbA1c, Fasting blood glucose (FBS), systolic and diastolic blood pressure in two groups of diabetic patients diagnosed in this program. The first group consisted of patients who received at least four visits by General Physician (GP) during one year after the diagnosis, and second group were patients who did not visited by GPs or received 1–3 visits.

**Results:**

After one year, 24.1% of patients did not receive any care, while 57.9% examined at least once a year. Among visited patients, 23.5% received 1–3 times medical care and 23.5% received four or more visits. HbA1c was significantly lowered in patients with appropriate care (four and more) compared with the non cared patients and patients with less than four cares.

**Conclusion:**

Appropriate number of visits for each patient by GPs is an effective glycemic control in diabetic patients. Although this study provides a framework for medical care in diabetes, how to take care of these patients depends on specific situation of each patient and should be determined for each of them individually.

## Background

According to the latest report by the World Health Organization (WHO), the prevalence of diabetes in the world will increase from 4% in 1995 to 5.4% in 2025. Further, the population of diabetics in the world will increase 122%, from 132 million in 1995 to 300 million in 2025 [1]. Unfortunately, the WHO projects that deaths resulting from diabetes will double between 2005 and 2030 [2]. The total number of excess deaths attributable to diabetes worldwide was estimated to be 3.96 million in the age group 20–79 years, 6.8% of global (all ages) mortality [3]. The excess global mortality attributable to diabetes in the year 2000 was estimated to be 2.9 million deaths, equivalent to 5.2% of all deaths. Excess mortality attributable to diabetes accounted for 2–3% of deaths in poorest countries and over 8% in the U.S., Canada, and the Middle East. In people 35–64 years old, 6–27% of deaths were attributable to diabetes [4].

Diabetics are at a 15-times greater risk of amputations than healthy individuals [5]. The findings from different population based studies for measuring prevalence of diabetic retinopathy suggested that the prevalence of more severe grades ranges from about 3% to 10% among diabetic patients [6,7]. The prevalence of blindness in diabetics has been reported in different studies and ranges from 0.4–1% [8,9]. The diabetes control and complications trial demonstrates that the general management of blood glucose levels is important in reducing the progress of diabetic retinopathy. Some studies have shown that the rate of nephropathy can be reduced by long-term control through annual laboratory tests and prompt treatment of micro-albuminuria [10].

Considering numerous long – term and serious complications of diabetes, several problems and restrictions of diabetes, such as ophthalmic, renal, vascular and nervous disorders, which lead to blindness, severe renal disease, amputation, Cerebrovascular accident (CVA) and Myocardial Infarction(MI)-, will occur if appropriate and prompt action concerning the prevention, control, and treatment of the disease is not taken. The primary aim of this study was to compare the HbA1c between those individuals with diabetes who have been visited and cared for by a family physician at least four times in the past twelve month in a rural health center with patients who have not been visited by their family physician within the past twelve months.

## Methods

### National program for diabetes control & prevention in Iran

To prevent and control diabetes and its complications in an early phase, Iran implemented the National Program for Diabetes Control and Prevention. This program was developed and implemented for the purposes of screening ‘individuals at risk’, and pregnant women. Individuals at risk consist of all people over 30 years old who have at least one risk factor as positive family history of diabetes, hypertension, overweight or obesity. Presently, the National Program for Diabetes Control and Prevention has been integrated into the national health system of Iran since 2004. The first stage of the program was implemented in rural areas of Iran and since then two rounds of screening have been held to cover non-covered portions of the country.

### Study population

In this study, 41 medical universities located within 30 provinces in Iran were responsible for providing health care services to the population of interest, among them 11universities were selected randomly. Among the diabetic patients, diagnosed in national program of diabetes in these universities, 1,423 type 2 diabetic patients were selected randomly proportional to the size of diagnosed diabetic patients in each selected university and followed from 2010 until 2011.

In the first step, patients were divided in two groups; 1-Patients who received any care in past 12 months by family physicians; and 2- Patients who did not receive any visits from a family physician. Then, we divided those patients who received care according to the numbers of medical visits during the first year following a diagnosis with diabetes. Since in the National Program for Diabetes Control and Prevention each GP has to visit diabetic patients four times per year, the patients were grouped depending on receiving one to three physician visits during the first year and those who were visited four or more times by their family general physicians. For each patient a questionnaire was completed.

For those patients who received care, we compared the fasting blood sugar (FBS), HbA1c, Body Mass Index (BMI), systolic and diastolic blood pressure, and mean arterial pressure in these two groups of patients at the time of screening and after one year of diagnosis with diabetes.

### Measurements

Data on age, height, and weight were collected by the questionnaire during the medical examination. BMI was calculated by dividing body weight in kilogram by the square of body height in meters and used to define subjects as normal (BMI below 25), overweight (BMI from 25 to 30), or obese (BMI above 30). Within one week after filling out the questionnaire, all patients were referred to the laboratory for measuring HbA1c and FBS. All tests were performed in the same laboratory and the chromatography method was used to measure HbA1c.

### Statistical analysis

All descriptive data are given as mean ± standard deviation, and as percentages when appropriate.

Analysis of covariance was done using general linear model, comparing three groups of diabetic patients to test difference in HbA1c adjusted for age, sex, BMI, and mean arterial pressure. Bonferoni post hoc test was applied to determine significant differences between specific groups on these variables. To compare the differences between variables in the time of diagnosis and 1 year after that, the paired t-test has been used.

The independent t-test was applied for comparison of FBS, BMI, and arterial pressure in two groups of patients; the patients who received appropriate care (at least 4 visits by GPs) and patients with no medical care. The statistical analyses were carried out with SPSS version 18.

## Results

Table 1 shows the baseline characteristics of the 1,423 diabetic patients obtained during the screening and following one year of diagnosis. Among the patients, 421(29.6%) were male and 1,002 (70.4%) were female. The mean age of the patients was 57.36 years (SD = 11.40) with a maximum age of 91 years. Information on the number of visits by general physicians was available on 1,134 patients. Among this group, 273(24.1%) patients did not receive any medical care, while 861(75.9%) were visited at least one time by GPs within the first year following a diagnosis of diabetes. Considering the number of visits, 266 (23.4%) patients were visited by their GPs one to three times and 595(75.9%) were visited four times or more. After one year of diagnosis, FBS decreased significantly (P-value ≤ 0.001), while mean arterial blood pressure rose dramatically (p-value ≤ 0.001) in all patients. Table 2 shows the comparison of HbA1c, FBS, BMI, and mean arterial blood pressure in two groups of patients after one year of disease diagnosis. The comparison of HbA1c and FBS adjusted for age, sex, and mean arterial pressure between the three groups of patients in regard to the number of cares showed a significant decrease in HbA1c in patients with four or more cares (p-value < 0.001). There was no statistical significant difference in HbA1c between those patients who received no care and those patients who were visited one to three times by GPs (Table 3). Table 4 shows the comparison of systolic blood pressure, diastolic blood pressure, BMI, and FBS at the time of screening and follow-up within one year in patients who have had appropriate care (four or more visits) by their GPs.

**Table 1:** baseline characteristics of 1423patients identified during screening and after 1 year

	**Mean ± Standard deviation**		**P-value**
	**Screening**	**One year later**	
BMI	28.25 ± 4.69	28.19 ± 4.68	1.60
Systolic Blood Pressure(mmHg)	124.63 ± 18.25	129.29 ± 21.40	<0.001
Diastolic Blood Pressure(mmHg)	75.51 ± 11.36	77.77 ± 11.86	<0.001
Mean arterial pressure	91.26 ± 12.17	94.25 ± 13.42	<0.001
Fasting blood sugar	204.30 ± 88.83	175.24 ± 69.71	<0.001

**Table 2:** comparison of HbA1c, FBS, BMI, and mean arterial blood pressure in patients with and without care by GPs in the end of first year of diagnosis of diabetes

	**Number**	**No care**	**Number**	**At least one care**	**p-value**
	**Mean ± SD**		**Mean ± SD**		
HbA1c	267	8.03 ± 2.25	858	7.78 ± 2.15	0.10
Fasting Blood Sugar	273	174.11 ± 69.86	861	172.04 ± 65.95	0.66
BMI	269	28.87 ± 4.89	857	27.79 ± 4.56	0.001
Mean arterial pressure	267	97.59 ± 14.01	858	93.64 ± 13.19	<0.001

**Table 3:** Mean and se HbA1c and FBS adjusted for age, sex, BMI, and mean arterial pressure in diabetic patients categorized by the number of care

	**No care (N = 273)**	**1-3 cares (N = 266)**	**4 cares and more(N = 595)**	**P-value**
Age	57 ± 0.66	57 ± 0.76	56 ± 0.46	0.42
Female (%)	61	71	69	0.36
HbA1c	7.9 ± 0.14	8.1 ± 0.14	7.3 ± 0.09	<0.001^*^
FBS	157 ± 4.20	162 ± 4.02	153 ± 2.7	0.01
Mean arterial pressure	93.55 ± 0.86	93.33 ± 0.82	92.89 ± 0.54	0.60

*statistically significant difference between 4 cares and more with no care (p-value = 0.01) and 1–3 cares and more (p-value = 0.001).

**Table 4:** comparison of mean systolic and diastolic blood pressure, BMI, and FBS in the time of diagnosis of diabetes and one year later in patients who have had appropriate care (4 and more) by general practitioner

	**Mean ± Standard deviation**		**P-value**
	**Screening**	**One year later**	
BMI	27.88 ± 4.43	27.78 ± 4.50	0.24
Systolic Blood Pressure(mmHg)	123.01 ± 16.93	126.81 ± 20.92	<0.001
Diastolic Blood Pressure(mmHg)	75.44 ± 11.14	76.98 ± 11.4	<0.001
Fasting blood sugar	200.88 ± 83.13	171.52 ± 4.32	<0.001

## Discussion

This study showed that the number of visits of diabetic patients by a GP plays an important role and desirable effect on controlling blood glucose and HbA1c, and every diabetic patient should be visited at least four times a year by their family physician. According to the authors knowledge this is the first study that evaluates the effect of number of medical care on diabetes control.

Tabrizi et al. in a review found that diabetes management programs with focus on regular visits are significantly related to better control of diabetes, reduced rates of diabetes complications, and reduced hospital admission. Other studies have also shown that improved of delivered care in both clinical and non-clinical areas increases quality of life and personal satisfaction and reduce the disease complications as well as the overall burden of type 2 diabetes.

One of the reasons that patients do not refer to their GP could be relate to quality of health care services. The service should focus on adequate manpower, personal and physical organization, financial resources and the rule of procedure. These factors have a significant relationship with quality of care and patients' satisfaction.

Another reason is related to the effective communication between doctor and patient. Physician – patient communication makes a significant difference to patient health outcomes. Physician education was demonstrated to affect the patients' emotional status, whereas patient education was demonstrated to affect physical health, level of function, blood pressure and blood glucose level. Good communication between physician – could encourage patients refer to the general physician in due time. Despite all been told the exact reason for not referring patient to their GP is not clear and should be studied in further research.

Hypertension and diabetes co-exist frequently resulting in significant mortality and morbidity. The prevalence of hypertension in diabetic patients is 1.5–2 times higher than in general population [11]. Significant increase in mean systolic and diastolic blood pressure in patients during the year of the study may be related to the fact that the physicians who being involved in the program have paid more attention to diagnosis and treatment of diabetes and miss other patients' problems. It is also might be related to macro- and micro-vascular complication of diabetes which makes hypertension treatment more difficult. Despite higher mean blood pressure of the patients in the end of the first year, diastolic and systolic blood pressure of the patients who had received at least four times visits by GPs (appropriate care) were significantly lower than the other patients. This finding emphasizes again the importance of medical care by family physicians. The mean blood pressure was increased in these patients when comparing with their blood pressure at the beginning of the study.

The important issue that needs to be considered in this regard is that when such a specific programs have been implemented and executed, it is also necessary to consider the patients' other health problems, and the health care providers especially GPs should be trained for appropriate management of co-existing problems.

One of the strengths of this study was the large study population which caused greater precision of the results. We also used the least number of medical laboratories possible for testing in order to reduce the variation of test results.

Based on the authors' knowledge there is no previous study to evaluate the relationship between the number of medical visits and blood glucose control in the diabetic patients. Masoudi-Alavia et.al in a Persian study entitled “Effect of design and implementation of health care model in diabetic patients” have declared that in this model, individuals, management system, and social security system are three systems interacting with each others that can cause good glycemic control in diabetic patients. They emphasize the management system as an important component for controlling the disease [12].

The limitation of the study was that the HbA1c was not measured in diabetic patients in the beginning of the study; therefore we compared the differences in HbA1c in patients who received 1 to 3 visits during a year after diabetes diagnosis with the patients who received at least 4 visits after one year. The patients were assigned in different groups randomly and there were no differences between the different comparison groups of patients.

## Conclusion

At least four visits by GPs in diabetic patients are necessary to effectively control the disease. Therefore, there is essential that different interventions to be used for encouraging patients to see their GPs regularly. The finding of this study could be used in planning and policy making for prevention and control of diabetes and its complication and use as a guide for interventional study in future in this field.

## Competing interests

The authors declare that they have no competing interests.

## Authors’ contributions

ARM participated in the study design and data acquisition, KE participated in the study design and data analysis, MH participated in data analysis and interpretation, and SMA participated in study design, data analysis and interpretation. All authors read and approved the final manuscript.

## Authors’ information

Ali-Reza Mahdavi is a senior expert in metabolic disease control and prevention and National Coordinator for Prevention of Blindness and Deafness at the Center for Non-communicable Disease Control in the Iranian Ministry of Health. he has contributed to such journals as the Journal of Hypertension, Archives of Iranian Medicine, and Obesity.

Koorosh Etemad is the Director of the Center for Non-communicable Disease Control in the Ministry of Health.

Muhiuddin Haider is a Research Associate Professor at the University Of Maryland School Of Public Health. His research focuses on strategies of behavior change, application of social marketing tools and communications capacity building. He has contributed to such journals as the Journal of Bangladesh Medical Research Bulletin, Journal of Health Communication, BRAC University Journal, and International Relations Forum Journal.

Seyed Mohammad Alavinia is the Director of the Endocrine and Metabolic Office at the Center for Non-communicable Disease Control in the Iranian Ministry of Health. He is also a Professor at North Khorasan University of Medical Sciences. He has contributed to such journals as the Scandinavian Journal of Work, Environment, and Health, International Archives of Occupational and Environmental Health, American Journal of Industrial Medicine. Seyed Mohammad Alavinia is the corresponding author and can be contacted at: malavinia2000@yahoo.com
